# Changed cerebral function and morphology serve as neuroimaging evidence for subclinical type 2 diabetic polyneuropathy

**DOI:** 10.3389/fendo.2022.1069437

**Published:** 2022-11-24

**Authors:** Lin-Mei Zhao, Xin Chen, You-Ming Zhang, Min-Li Qu, Dinesh Selvarajah, Solomon Tesfaye, Fang-Xue Yang, Chu-Ying Ou, Wei-Hua Liao, Jing Wu

**Affiliations:** ^1^ Department of Endocrinology, Xiangya Hospital, Central South University, Changsha, China; ^2^ Department of Radiology, Xiangya Hospital, Central South University, Changsha, China; ^3^ Department of Radiology and Radiological Sciences, Johns Hopkins Hospital, Johns Hopkins University School of Medicine, Baltimore, MD, United States; ^4^ National Clinical Research Center for Geriatric Disorders, Xiangya Hospital, Central South University, Changsha, China; ^5^ Hunan Engineering Research Center for Obesity and its Metabolic Complications, Xiangya Hospital, Central South University, Changsha, Hunan, China; ^6^ Diabetes Research Unit, Sheffield Teaching Hospitals NHS Foundation Trust, Sheffield, United Kingdom; ^7^ National Engineering Research Center of Personalized Diagnostic and Therapeutic Technology, Xiangya Hospital, Central South University, Changsha, China

**Keywords:** type 2 diabetic polyneuropathy, preclinical, neuropathic pain, cortical volume, functional connectivity, resting-state functional MRI

## Abstract

**Introduction:**

Central and peripheral nervous systems are all involved in type 2 diabetic polyneuropathy mechanisms, but such subclinical changes and associations remain unknown. This study aims to explore subclinical changes of the central and peripheral and unveil their association.

**Methods:**

A total of 55 type-2 diabetes patients consisting of symptomatic (n = 23), subclinical (n = 12), and no polyneuropathy (n = 20) were enrolled in this study. Cerebral morphology, function, peripheral electrophysiology, and clinical information were collected and assessed using ANOVA and post-hoc analysis. Gaussian random field correction was used for multiple comparison corrections. Pearson/Spearman correlation analysis was used to evaluate the association of the cerebral with the peripheral.

**Results:**

When comparing the subclinical group with no polyneuropathy groups, no statistical differences were shown in peripheral evaluations except amplitudes of tibial nerves. At the same time, functional connectivity from the orbitofrontal to bilateral postcentral and middle temporal cortex increased significantly. Gray matter volume of orbitofrontal and its functional connectivity show a transient elevation in the subclinical group compared with the symptomatic group. Besides, gray matter volume in the orbitofrontal cortex negatively correlated with the Neuropathy Symptom Score (r = -0.5871, p < 0.001), Neuropathy Disability Score (r = -0.3682, p = 0.009), and Douleur Neuropathique en 4 questions (r = -0.4403, p = 0.003), and also found correlated positively with bilateral peroneal amplitude (r > 0.4, p < 0.05) and conduction velocities of the right sensory sural nerve(r = 0.3181, p = 0.03). Similarly, functional connectivity from the orbitofrontal to the postcentral cortex was positively associated with cold detection threshold (r = 0.3842, p = 0.03) and negatively associated with Neuropathy Symptom Score (r = -0.3460, p = 0.01).

**Discussion:**

Function and morphology of brain changes in subclinical type 2 diabetic polyneuropathy might serve as an earlier biomarker. Novel insights from subclinical stage to investigate the mechanism of type 2 diabetic polyneuropathy are warranted.

## Introduction

Type 2 diabetic polyneuropathy (DPN) is one of the most common complications of diabetes. It could lead to various sensorimotor deficits (1), even significant disability and diminished quality of life (2). Pharmacotherapy remains the mainstay of treatment but often has poor efficacy and is limited by unwanted side effects (3). Early intervention is proposed as the most promising method to improve treatment outcomes and prevent DPN and its cascade of devasting sequelae (4). Thus, improving insights into the early mechanism may improve patient management.

Peripheral and central nervous systems have been demonstrated to be involved in the mechanism of clinical DPN (5). Tight associations of central with peripheral changes were also unveiled in many previous studies (6–12). However, whether a connection and to what extent exists between subclinical central and peripheral neuropathy remain controversial. Regarding the earliest stages in diabetes patients without neuropathy, some researchers insist that cortical atrophy is impacted by independent diabetes effects (13, 14). Early central conduction abnormalities were also found independent of peripheral nerve changes (15). In contrast, early alterations on the spinal cord (16) during subclinical DPN and its tight association with peripheral severity demonstrated a subclinical connection between the spinal cord and peripheral, increasing attention on the subclinical stage. There is a possibility from these studies that central and peripheral neuropathy with different stages suffers from separate pathological connections. Further support for this theory comes from studies showing a discrepancy in cerebral functional and structurally dynamic change and even plasticity (17) at different severity of DPN, and dysfunction along somatosensory efferent ways relies on the stage of DPN (18) has also been proven. Therefore, a key point of truly unveiling relationships between central and peripheral alterations lies in the enrollment of different stages of DPN, and no similar studies have been reported.

In this study, we underscore the importance of a subclinical stage of DPN. We performed cerebral structural and functional analysis and examined multi-phase central and peripheral nervous system alterations. We then aim to explore the possible connections between central and peripheral neuropathy in different stages and determine how cerebral function and morphology react to peripheral changes.

## Methods

All subjects acquired written informed consent before attending the study and got prior approval from the Medical Research Ethics Committee of Xiangya Hospital, Central South University (201709981).

### Subjects

**Figure 1:**
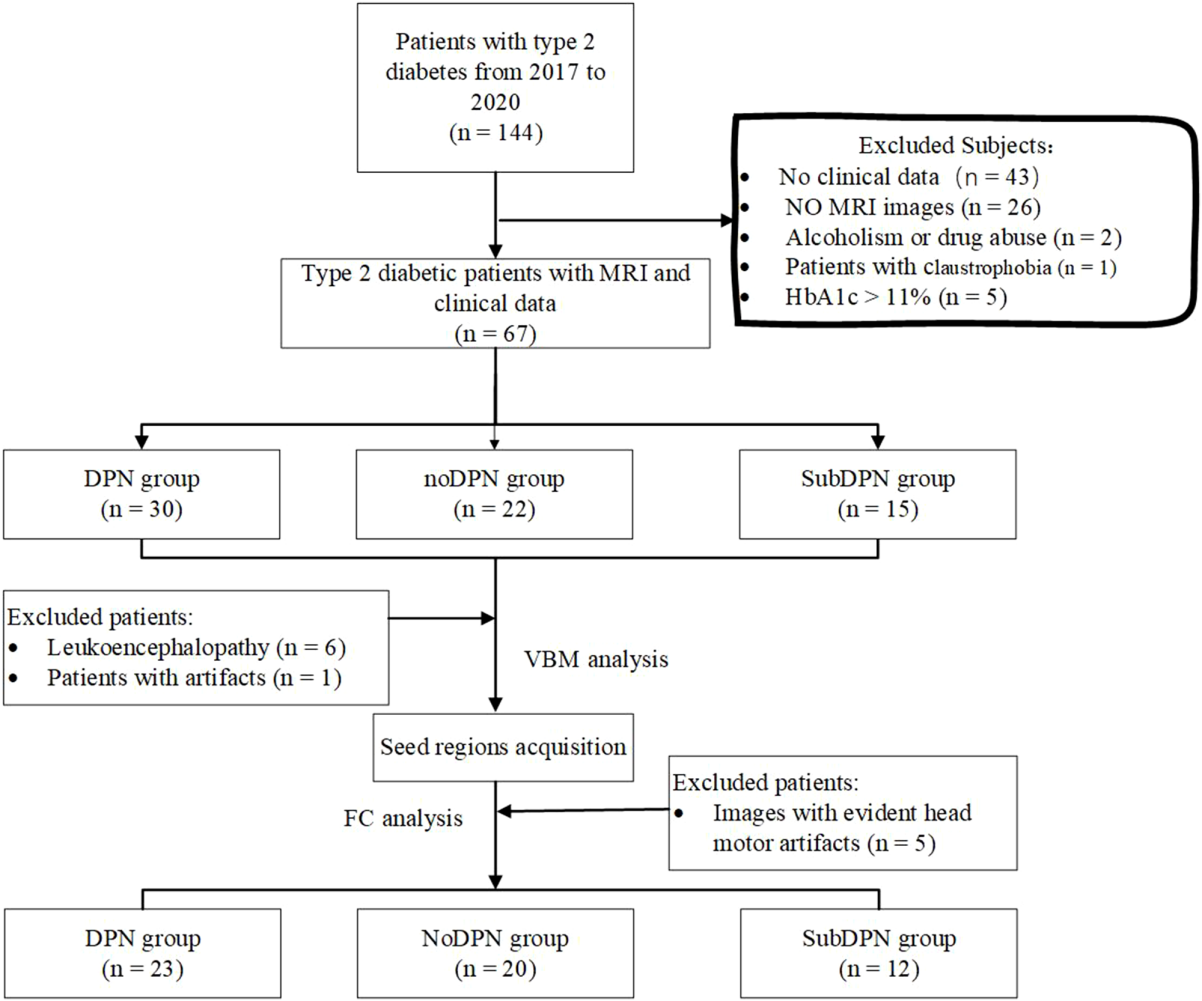
Flowchart diagram for subject selection. DPN, type 2 diabetic polyneuropathy; subDPN, subclinical type 2 diabetic polyneuropathy; noDPN,type 2 diabetes without polyneuropathy; MRI, magnetic resonance imaging; VBM, voxel-based morphometry; FC, functional connectivity.

Patients with type-2 diabetes were recruited from the Department of endocrinology at Xiangya Hospital, Central South University in China. The inclusive criteria were: 1) 3 years after a type-2 diabetes diagnosis was confirmed, 2) right-handedness, and 3) age between 18 and 70 years. We excluded subjects for the following criteria: 1) HbA1c (%, mmol/mol)was over 11%, 2) deficiency of clinical or imaging data, 3) evident intracranial lesions such as retinopathy, cerebrovascular disease, psychiatric diseases, brain trauma, tumors, white matter aberrance, or any other primary intracranial disease and history of surgery, 4) alcoholism or drug abuse, 5) patients with claustrophobia or other contraindication to MRI, and 6) images with artifacts (**Figure 1**).

### Clinical and neuropathic assessments

Participants in this study underwent routine clinical evaluation, such as gender, age, body mass index (BMI), diabetic duration, HbA1c [%(mmol/mol)], and years of education.

All patients were examined with neuropathological assessments: 1) standard questionnaires such as the neuropathy symptom score (NSS), neuropathy disability score (NDS), and douleur neuropathique 4 questions (DN4). 2) neurophysiology testing consisting of quantitative sensory testing (QST, TSA II) and nerve conduction testings (NCTs, Nihon Kohden MEB-9400). Specifically, QST mainly detected cold and warm detection thresholds (CDT/WDT) from the dorsal surface of the bilateral foot with the standard technique (19). NCTs were performed at a stable skin temperature of 31°C and a room temperature of 24°C. The nerves measured were: 1) sensory nerve conduction velocities (SCV) and amplitudes of sural sensory nerve, 2) motor nerve conduction velocities (MCV) and amplitudes of common peroneal and tibial motor nerves.

Subjects with type-2 diabetes were divided into three groups based on neuropathy assessments above (20, 21): 1) type-2 diabetes without polyneuropathy (noDPN) group: subjects of no clinical symptoms and signs and abnormal neurophysiological assessments. 2) subclinical type 2 diabetic polyneuropathy (subDPN) group: subjects with neither clinical symptoms nor signs but at least one abnormality on neurophysiological assessments. 3) type 2 diabetic polyneuropathy group: subjects with clinical symptoms or/and signs and at least two abnormalities of neurophysiological assessments.

### Image acquisition

All image data were collected from the same 3.0 T MRI scanners (Magnetom Prisma, Siemens, Germany) with a 64-channel head coil. The acquisition sequences of anatomy data were three-dimensional T1-weighted MRI scans through magnetization prepared rapid acquisition gradient-echo sequence with the following parameters. Thickness/gap:1.0/0 mm, repetition time: 2,300 ms, echo time: 2.98 ms, inversion time: 900 ms, 176 sagittal slices, the field of view: 256 mm × 256 mm, matrix: 256 × 256, flip angle: 9°, voxel size: 1.0 mm × 1.0 mm × 1.0 mm, sequence scan time: 5.09 min.

The resting-state functional data were acquired from echo-planar imaging sequences with the following parameters: repetition time: 2,000 ms, echo time: 30 ms, volumes: 240, slice thickness: 2, flip angle: 66°, in-plane pixel dimensions: 2.34 mm × 2.34 mm, total acquisition time: 8.15 min. Subjects were all asked to keep their eyes closed and awake without thinking about anything. Besides, the study collected T2-weighted fluid-attenuated inversion recovery sequence to exclude evident cerebral lesions.

### Image preprocessing analysis

Before structural and functional data preprocessing, all data were transferred to NIfTI format. Structural data were preprocessed in the VBM 8 toolbox (http://dbm.neuro.uni-jena.de/vbm8/) embedded with Statistical Parametric Mapping 8 (SPM8, Wellcome Department of Cognitive Neurology, London, UK. www.fil.ion.ucl.ac.uk/spm/software/spm8). The preprocessing steps included segmentation, normalization, and smoothness. These images were split into gray matter, white matter, and cerebrospinal fluid with the segmentation algorithm. Diffeomorphic anatomical registration through exponentiated lie algebra (DARTEL) (22) algorithm was used for spatial coregistration to the Montreal neurological institute(MNI) space, and images with 1.5 mm × 1.5 mm × 1.5 mm voxel were acquired for subsequent steps. The smoothed images with a Gaussian kernel of 8 mm full-width at half-maximum were acquired for subsequent analysis. All those steps were finished in the pipeline “Estimate & Write” of VBM8.

The resting-state functional MRI data were preprocessed with Data Processing & Analysis for Resting-State Brain Imaging (DPABI, http://rfmri.org/dpabi) based on SPM8 and Matlab R2013b (23). After the first ten time points were removed, the subsequent preprocessing steps were as follows: slice timing, realignment, spatially normalization, smoothness, regression, filter, and detrending. Specifically, slice timing was carried out with corresponding slice orders and the reference order. Realignment requires head motion parameters displacement, mainly translation and rotation computed by a linear transformation with a six-parameter (rigid-body), within (x,y, or z-direction) 2 mm and 2 degrees (24). Then realigned images were normalized to MNI space with 3 mm × 3 mm × 3 mm voxel using DARTEL registration (22). Smoothness was done with a Gaussian kernel of 6 mm full-width at half-maximum, and detrending was performed to eliminate the thermal noise caused by the MRI machine. In addition, to avoid the effects of physiological low-frequency and high-frequency noise, we applied a filter to ensure the frequency was at 0.01- 0.08 Hz. Besides, other covariate nuisances were regressed, including white matter and cerebrospinal fluid signal, six head motion parameters, and global mean signal. The preprocessed images were applied for subsequent analysis.

### Image analysis

One-way analysis of variance analysis (ANOVA) and *post-hoc* analysis was applied to compare differences in gray matter volume (GMV) among three groups, with Gaussian random field (GRF) multiple comparisons correction at voxel *p* < 0.005 and cluster *p* < 0.05. The brain areas with differences in cortical volume were selected as seed regions for subsequent functional connectivity analysis.

Whole-brain voxel-based functional connectivity (FC) was performed by Pearson correlation between the seed regions and the rest of the brain regions at a voxel level using DPABI. The correlation coefficients were then normalized to Z-scores using Fisher to Z transform. ANOVA analysis was applied to compare FC among groups. *Post-hoc* analysis was used to compare in pairs.

Besides, the specific values of FC and gray matter volumes were extracted from ROIs using the DPABI pipeline “ROI Signal Extractor” for subsequent correlation analysis.

### Statistical analysis

Statistical analyses were performed in SPSS 26.0 (SPSS Inc., Chicago, IL) and DPABI. Continuous variables were shown as the mean and standard deviation, while the discrete variables were presented as numbers (percentages). ANOVA and *post-hoc* analysis were used for comparisons of group differences. Specifically, in SPSS, the homogeneity of variance was analyzed with Levene analysis. Besides, those with homoscedasticity underwent the Least Significance Difference test. In contrast, variables with heteroscedasticity underwent the nonparametric Kruskal-Wallis test. The significance level for the comparisons was a two-tailed *p*-value < 0.05.

In DPABI, each contrast was entered into ANOVA and *post-hoc* analysis regarding sex, age, and intracranial volume as covariates. Results were first thresholded at voxel-wise *p* < 0.005 and then corrected at the cluster level *p* < 0.05 for multiple comparisons using GRF correction.

Pearson and Spearman correlation analysis, with corresponding normal and non-normal distribution circumstances, was used to examine associations of parameters from brain structure and function with individual attributes of clinical assessments. The analysis was completed on SPSS 26.0. A two-tailed *p*-value less than 0.05 was conceived as a significance level.

## Results

### Demographic findings

The study cohort consisted of 20 type 2 diabetic patients with no DPN, 12 type 2 diabetic patients with subclinical DPN, and 23 type 2 diabetic patients with DPN. Gender (*p* = 0.944), education year (*p* = 0.053), BMI (*p* = 0.454), HbA1c (*p* = 0.624), and diabetes duration (*p* = 0.080) (**Table 1**) among the three groups show no statistical significance. The mean age of DPN patients was (56.96 ± 9.49) years, older than noDPN participants by (48.85 ± 9.06) years (*p* = 0.004).

**Table 1:** Results of demographic characteristics and intracranial volumes.

	noDPN (n = 20)	subDPN (n = 12)	DPN (n = 23)
**Gender**			
**Male**	14 (70.0)	9 (75.0)	17 (73.9)
**Female**	6 (30.0)	3 (25.0)	6 (26.1)
**Age (years)**	48.85 ± 9.06	54.42 ± 7.01	56.96 ± 9.49^**^
**Education (years)**	13.18 ± 3.43	14.25 ± 1.98	11.00 ± 3.79
**BMI (kg/m** ^2^ **)**	25.08 ± 2.75	23.52 ± 3.61	23.89 ± 3.76
**HbA1c [%(mmol/mol)]**	7.73 ± 2.29	8.46 ± 1.35	7.85 ± 2.03
**Disease duration (years)**	4.40 ± 3.57	7.61 ± 5.09	8.55 ± 6.26
**DN4**	0.17 ± 0.39	0.20 ± 0.42	3.90 ± 2.00^***†††^
**NSS**	0.53 ± 1.13	0.33 ± 0.65	6.61 ± 1.53^***†††^
**NDS**	0.20 ± 0.41	0.55 ± 1.04	2.52 ± 1.95^***††^
**L_PN_MCV (m/s)**	47.83 ± 4.39	45.35 ± 3.07	41.69 ± 6.48^**^
**L_PN_Am (mV)**	7.64 ± 2.69	6.02 ± 2.28	4.44 ± 2.47^**^
**R_PN_MCV (m/s)**	48.86 ± 4.17	46.56 ± 5.20	42.29 ± 6.53^**†^
**R_PN_Am (mV)**	7.03 ± 2.93	6.59 ± 3.15	4.64 ± 2.45^*^
**L_TN_MCV (m/s)**	47.92 ± 4.71	45.89 ± 5.45	40.88 ± 7.34^**†^
**L_TN_Am (mV)**	20.24 ± 5.18	16.62 ± 4.34	13.96 ± 8.12^**^
**R_TN_MCV (m/s)**	48.58 ± 5.71	45.83 ± 4.59	41.73 ± 6.53^**^
**R_TN_Am (mV)**	21.11 ± 5.59	15.08 ± 5.04^**^	12.46 ± 5.67^***^
**L_SN_SCV (m/s)**	56.28 ± 6.99	52.77 ± 3.43	49.08 ± 8.18^**^
**L_SN_Am (uV)**	18.66 ± 10.7	12.18 ± 8.71	10.85 ± 8.65^*^
**R_SN_SCV (m/s)**	54.66 ± 5.22	52.73 ± 4.06	48.22 ± 4.99^**†^
**R_SN_Am (uV)**	18.24 ± 11.02	12.11 ± 5.39	9.79 ± 7.16^**^
**L_CDT (°C)**	27.05 ± 3.58	25.77 ± 10.25	28.26 ± 2.99
**R_ CDT (°C)**	27.78 ± 3.01	25.94 ± 6.7	26.93 ± 6.39
**L_ WDT (°C)**	39.94 ± 3.48	41.88 ± 5.06	40.79 ± 3.54
**R_WD T (°C)**	41.80 ± 4.41	42.25 ± 4.67	40.25 ± 3.07
**TIV (cm** ^3^ **)**	1403.78 ± 124.78	1424.42 ± 130.81	1421.49 ± 112.66
**gGMV (cm** ^3^ **)**	640.07 ± 52.21	657.68 ± 50.64	634.40 ± 64.58
**gWMV (cm** ^3^ **)**	525.48 ± 65.03	521.44 ± 70.52	519.32 ± 52.94

Values were given as a number (ratio) for discrete parameter and mean and standard deviation for continuable parameters. ^*^ *p* < 0.05, ^**^ *p* < 0.01, ^***^ *p* < 0.001 compared with noDPN groups, ^†^ *p* < 0.05, ^††^ *p* < 0.01, ^†††^ *p* < 0.001 compared with subDPN group.

BMI, body mass index; DN4, Douleur Neuropathique en 4 questions; NSS, Neuropathy Symptom Score; NDS, Neuropathy Disability Score; L, left; R, right; MCV, motor conduction velocity; SCV, sensory nerve conduction velocities. Am, amplitude; PN, the common peroneal nerve; TN, tibial nerve; SN, sural nerve; CDT, cold detection threshold; WDT, warm detection threshold; TIV, intracranial volume;gGMV, global matter volume; gWMV, global white matter volume; DPN, type 2 diabetic polyneuropathy; subDPN, subclinical type 2 diabetic polyneuropathy; noDPN, type 2 diabetes without polyneuropathy.

### Neuropathic assessments results

Bilateral cold and warm thresholds detection with no statistically significant were observed among individuals (**Table 1** **).** When comparing the DPN group with other groups, DN4, NSS, and NDS in subjects were highest, while MCV of right peroneal and left tibial nerves and SCV of right sural nerves were lowest (*p* < 0.05). Nevertheless, when comparing the subDPN group with the noDPN group, a lower amplitude of the right tibial nerve was the only significant one observed (*p* = 0.006), and other parameters showed no significant difference. When comparing the clinical DPN group with the noDPN group, amplitudes of bilateral common peroneal nerves, left tibial nerves, and bilateral sural sensory nerves were significantly lower. Lower MCV of the left common peroneal nerve, right tibial nerve, and SCV of the left sural nerve were all also observed (*p* < 0.05) in the clinical DPN versus the noDPN group (**Table 1** and **Figure S1**).

### Image findings

**Figure 2:**
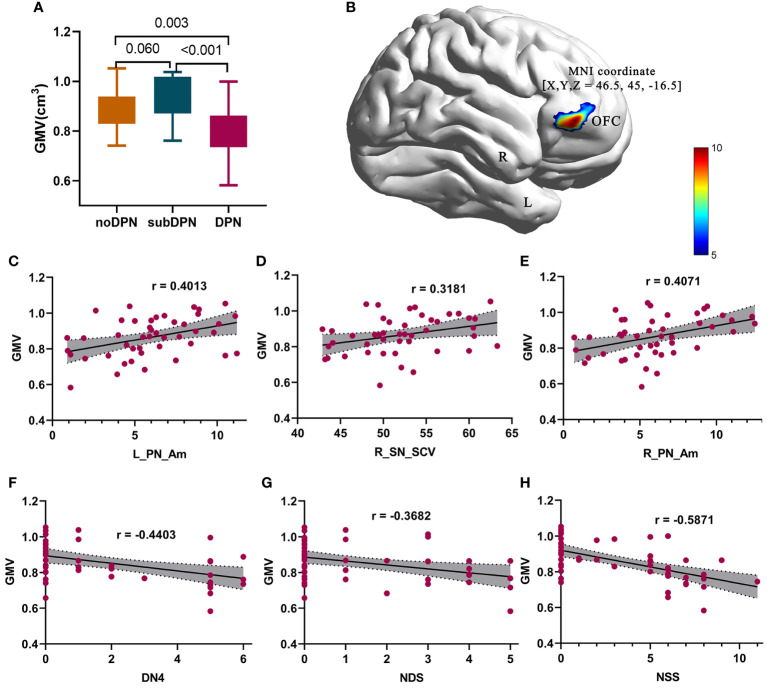
Gray matter volume difference presentation and related correlations results. **(A)** *post-hoc* results of regional gray matter volume in a bar graph. **(B)** significant difference of brain regions within the orbitofrontal cortex. **(C–E) **positive correlations of GMV with L_PN_Am **(C)**, R_SN_SCV **(D)**, and R_PN_Am **(E)** were shown in points and lines. **(F-H)** negative correlations of GMV with DN4 **(F)**, NDS **(G)**, and NSS **(H)** were shown in points and lines. The color bar denotes the corresponding T-values. DPN, type 2 diabetic polyneuropathy; subDPN, subclinical type 2 diabetic polyneuropathy; noDPN,type 2 diabetes without polyneuropathy; GMV, gray matter volume; OFC, the orbitofrontal cortex; L, left; R, right; NSS, the Neuropathy Symptom Score; DN4, Douleur Neuropathique en 4 questions; NDS, Neuropathy Disability Score; SCV, sensory nerve conduction velocities. Am, amplitude; PN, the common peroneal nerve; SN, sural nerve.

There was no significant difference in intracranial volume, global GMV (gGMV) and white matter volume (gWMV) (**Table 1**). GMV within the right orbitofrontal cortex (OFC) showed statistically significant among three groups (GRF: voxel *p* < 0.005, cluster *p* < 0.05), with 523 voxels and peak MNI coordinate X/Y/Z: 46.5/45/-16.5 (**Figure 2B**). A transient increase of GMV within right OFC was found in subclinical subjects compared with clinical subjects (*p* < 0.001) and noDPN patients (*p* = 0.06). Decreased GMV in the DPN group was also observed compared with the noDPN group (**Figure 2A**).

**Figure 3:**
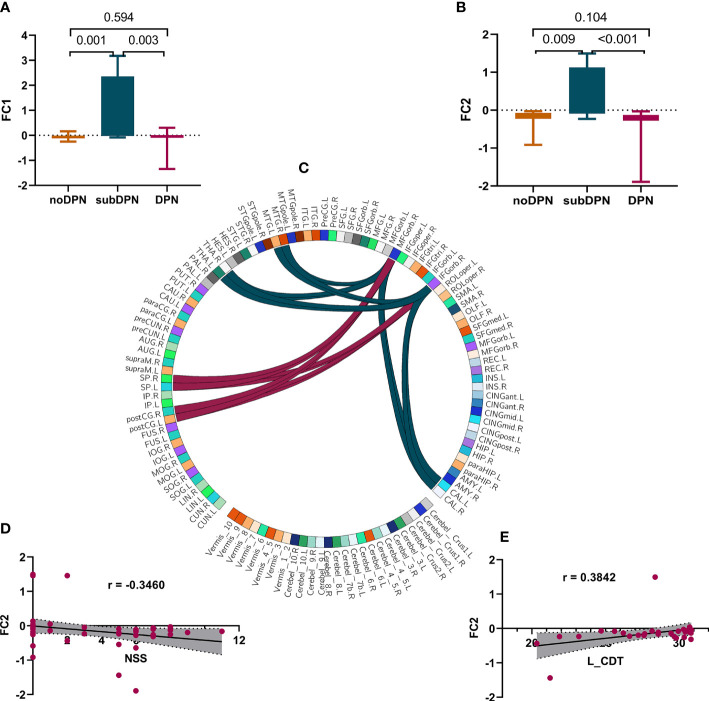
Presentations of functional connectivity differences among groups and related correlations. **(A, B)**. *post-hoc* results of FC1 **(A)** and FC2 **(B)**. **(C)** a sketch diagram denoted a significant difference in functional connectivity between cerebral regions. The dark green and red link lines denote FC1 and FC2, respectively. **(D, E)**. correlations of FC2 with NSS**(D)** and left CDT **(E)**. FC, functional connectivity values after fisher-Z transformation; FC1. connectivity of OFC with bilateral MTG/THA/CAL. FC2. connectivity of OFC with bilateral postCG/SP. L, left; R, right. OFC, the orbitofrontal cortex; MTG, middle temporal gyrus; THA, thalamus; CAL, calcarine gyrus; postCG, the postcentral gyrus; SP, superior parietal gyrus; NSS, the Neuropathy Symptom Score; CDT, cold detection threshold; DPN, type 2 diabetic polyneuropathy; subDPN, subclinical type 2 diabetic polyneuropathy; noDPN,type 2 diabetes without polyneuropathy.

The voxel-wise functional connectivity from right OFC to the bilateral middle temporal gyrus (MTG)/calcarine/thalamus and bilateral postcentral gyrus/superior parietal cortex (**Figure 3C**) showed a statistically significant increase in the subDPN group in comparison to the other two groups (GRF: voxel *p <*0.005, cluster *p <*0.05). Specifically, positive connectivity in the subclinical group was observed (**Figures 3A, B**).

### Correlation results

Negative correlations of GMV of the OFC could be found with NSS (r = -0.5871, *p* < 0.001) (**Figure 2H**), NDS (r = -0.3682, *p* = 0.009) (**Figure 2G**), and DN4 (r = -0.4403, *p* = 0.003) (**Figure 2F**). In contrast, positive correlations of GMV of OFC with bilateral peroneal amplitude [L: r = 0.4013, *p* = 0.0063 (**Figure 2C**); R: r = 0.4071, *p* = 0.0055 (**Figure 2E**)] and SCV of the right sensory sural nerve (r = 0.3181, *p* = 0.03**) (** **Figure 2D**) were also observed. At the same time, functional connectivity of right OFC with bilateral postcentral gyrus/superior parietal gyrus was positively associated with CDT (r = 0.3842, *p* = 0.03) (**Figure 3E**) and negatively associated with NSS (r = -0.3460, *p* = 0.01) (**Figure 3D**).

## Discussions

The study examined cerebral changes in subjects with a subclinical phase of DPN, which is an extension of previous studies of DPN. The novel observation from our study included two folds: a. Compared with the noDPN group, subjects of the subDPN group presented significant functional alterations in the brain, and a transient elevation exists in both cerebral morphology and function. b. Cerebral morphology changes were related to peripheral motor and sensory abnormalities, while cerebral functional changes were only associated with peripheral sensory abnormality. Closer examination also suggested cortical function alterations were primarily in regions more related to sensory perception and structure regions located more related to neuropathic pain. This study demonstrated sensitivity in brain function changes during a subclinical stage and suggested that the brain would also play a critical role in an early phase of DPN.

Early sensory function impairment is always a major concern for DPN. QST is a quantitative method of assessing sensations such as temperature, vibration and stimulation with a limit. Similar to a previous study (25), the QST results in our study showed no significant difference when comparing clinical DPN with the other two groups. Besides, as a psychosomatic test, QST results can be affected by many confusion factors (26), and differences in sensory tolerance might also contribute to differences in results declared by other studies (27, 28). In contrast, the cerebral morphology and function results in this present study showed an underlying potential sensitive biomarker.

Minor structural and functional changes in the cerebra of clinical DPN have been well confirmed for many years, particularly for gray matter volume and functional connectivity (6, 8, 17). However, subjects with subclinical DPN were not included in those previous studies. A principal strength of this study was the inclusion of a subclinical cohort, which was a salient complement to previous studies. Based on subclinical DPN cohorts, we found a gray matter volume reduction within the right OFC in subjects with DPN compared with subclinical and noDPN subjects. The OFC is classically involved in the emotion, reward, and cognitive processes such as decision-making (29). Notably, the involvement of OFC in the sensory process (30) and affective components of pain has been extensively demonstrated (31), yet little is known regarding its role in neuropathy. This study in DPN first directly reveals a possible correlation of OPC with neuropathic pain indexes like NSS, NDS and DN4, indicating its important role in neuropathic pain. This idea is further supported by a study that focused on the modulatory role of OFC in the nociception process, which reported projection from OFC to the ventrolateral periaqueductal gray matter (vlPAG) (32). Given that the vlPAG can receive and modify the information from the cortex, most previous studies found the connectivity of the primary somatosensory cortex (S1) with vlPAG. However, these studies are from a clinical cohort, and when subclinical DPN subjects were enrolled in this study, OFC-S1/superior parietal connectivity was also observed. Hence, we postulated that there might be S1-OFC-vlPAG circuits engaged in neuropathic pathogenesis. Nevertheless, further studies were demanded to unveil complete circuits from the periphery to the spinal cord and then the brain, as the inability of this study to access a simultaneous spinal-brain MRI.

Intriguingly, the GMV and functional connectivity tended to be consistently a transient elevation during the subclinical phase. Increased activation of somatosensory cortices appeared in the early neuropathic pain (33) while not observable in the late phase, and such changes were considered a regulation of the neuron circuit (34). Given the physical and spatial close relationship between brain structure and function (35), this study may give a structural explanation that variations of functional connectivities were accompanied by structural reorganization of DPN (17). Furthermore, one trait of the brain is that greater GMV and functional connectivities were associated with better intravenous lidocaine response (36). Our findings of subclinical DPN suggest that the subclinical phase may play a significant role in paresthesia treatment response. This could be further supported by results from the present study that showed significant correlations of GMV and functional connectivities with sensory parameters like CDT and SCV of the sural nerve.

Several limitations in this present study need to be addressed. Firstly, this study cannot interpret the causality of the brain and the peripheral nerves due to a cross-sectional investigation being designed. However, enrollment of DPN patients with different severity may help establish a better understanding of the disease progression and prepare for the subsequent longitudinal investigation. In addition, the correlations between cognition and cerebral alteration were not performed as this was not assessed. Although cognition assessment compromised an important part of DPN, in this study, we care more about the early brain structure and function changes and their relationship with peripheral nerves. More detailed cognition data are expected in future studies to determine early cognition alterations and their associations with the brain during the early phase of DPN. Lastly, this exploratory clinical trial has a relatively small sample size with inter-group differences, limiting the ability to test the results of a subgroup of clinical DPN (such as the painful and painless group). Future studies of a larger cohort would be warranted to produce a stable and comprehensive performance.

In conclusion, this present study provides evidence of significantly altered brain morphology and function in mild peripheral neuropathy at the subclinical stage. These discrepant findings suggest a critical role of central regulation on DPN and give a novel underlying mechanism. Furthermore, early cerebral function changes may interact more with sensory deficits. In contrast, cerebral morphology reacts to both motor and sensory abnormalities, giving novel insights into a specific biomarker for a different stage.

## Data availability statement

The data supporting the findings of this present study are available from the corresponding authors upon reasonable request.

## Ethics statement

The studies involving human participants were reviewed and approved by Medical Research Ethics Committee of Xiangya Hospital, Central South University. The patients/participants provided their written informed consent to participate in this study.

## Author contributions

W-HL, JW contributed to the study conceptualization. L-MZ and Y-MZ contributed to the development of methodology and data analysis. L-MZ, XC, C-YO, and F-XY contributed to data collection and image quality insurance, L-MZ and XC contributed to writing the manuscript. W-HL, JW, DS, and ST contributed to reviewing and editing the manuscript. W-HL and JW contributed to the funding acquisition. W-HL and JW were guarantors of this work, had full access to all the data, and took responsibility for the integrity and accuracy of data. All authors contributed to the article and approved the submitted version.

## Funding

This study was supported by the National Natural Science Foundation of China, Grant/Award Numbers: 82170849, 82071894, 91959117; Projects of International (Regional) Cooperation and Exchanges of the National Natural Science Foundation of China, Grant/Award Numbers: 81911530222; Science and Technology Innovation Program of Hunan Province, Grant/Award Numbers: 2020RC4007, the Key Research & Development Plan, Hunan, China, Grant/Award Numbers: 2020SK2066, Science and Technology Program of Changsha, China, Grant/Award Numbers: kh2003010; National Clinical Research Center for Geriatric Disorders Foundation, Grant/Award Number 2021LNJJ04.

## Acknowledgments

The authors thank all subjects who joined this study. The authors acknowledge the support from all team members, students, and technical specialists.

## Conflict of interest

The authors declare that the research was conducted in the absence of any commercial or financial relationships that could be construed as a potential conflict of interest.

## Publisher’s note

All claims expressed in this article are solely those of the authors and do not necessarily represent those of their affiliated organizations, or those of the publisher, the editors and the reviewers. Any product that may be evaluated in this article, or claim that may be made by its manufacturer, is not guaranteed or endorsed by the publisher.
