# Supplementary material for: Changed cerebral function and morphology serve as neuroimaging evidence for subclinical type 2 diabetic polyneuropathy
Source: Front Endocrinol (Lausanne). 2022 Nov 24;13:1069437. doi: 10.3389/fendo.2022.1069437 (PMC9729333; doi:10.3389/fendo.2022.1069437)
Supplement: Supplementary file 1 [file DataSheet_1.docx]

Supplementary Material

# Supplementary Figures and Tables

## Supplementary Figures


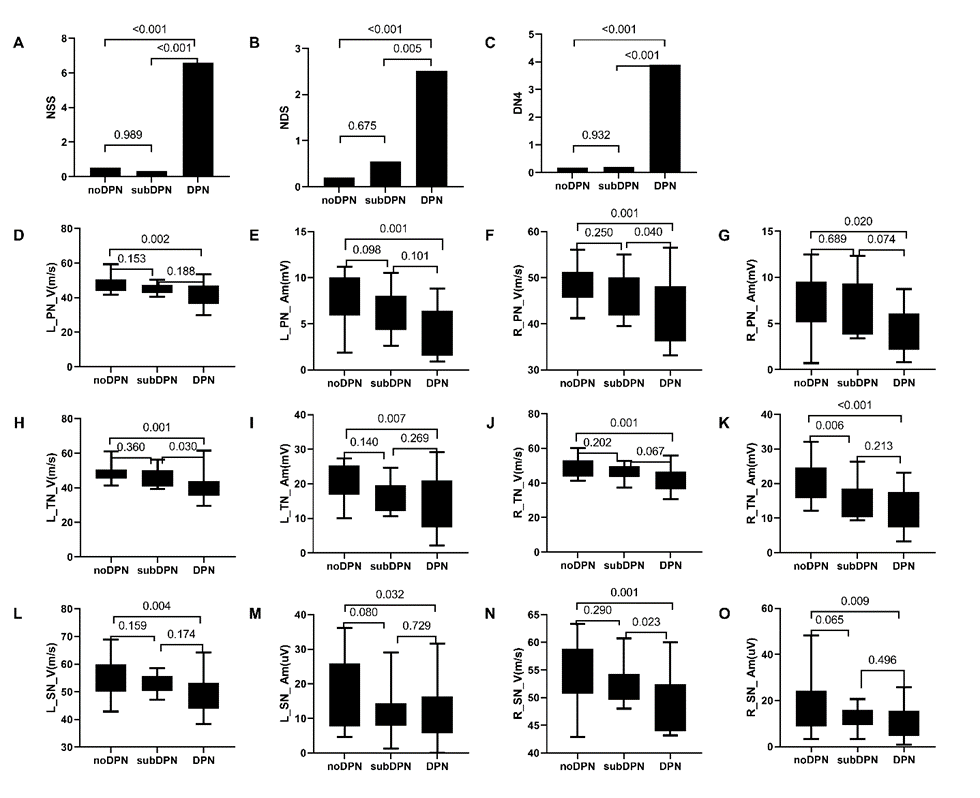


**Supplementary Figure 1.** Bar diagram of neurophysiologic assessments from post-hoc results. A-C, post-hoc results of NSS (A), NDS(B), and DN4(C). D-G, post-hoc results of amplitudes (E-left, G-right)and MCV (D-left, F-right) of bilateral peroneal nerves. H-K, post-hoc results of amplitudes (I-left, K-right)and MCV (H-left, J-right) of bilateral tibial nerves. L-O, post-hoc results of amplitudes (M-left, O-right)and SCV (L-left, N-right) of bilateral sural sensory nerves. NSS, the Neuropathy Symptom Score; NDS, Neuropathy Disability Score, DN4, Douleur Neuropathique 4 questions, MCV, motor conduction velocity, SCV, sensory nerve conduction velocities, DPN, type 2 diabetic polyneuropathy; subDPN, subclinical type 2 diabetic polyneuropathy; noDPN,type 2 diabetes without polyneuropathy
